# Evidence for a Griffiths Phase to Cluster Spin Glass Transition in the La_2/3_Sr_1/3_(Mn_1‐3_ *_x_* Al_2_ *_x_* Ti*_x_*)O_3_ System

**DOI:** 10.1002/advs.202408517

**Published:** 2024-10-14

**Authors:** Ruie Lu, Yuanchao Ji, Yu Wang, Xiaoqin Ke, Fanghua Tian, Chao Zhou, Yin Zhang, Chang Liu, Sen Yang, Xiaobing Ren, Xiaoping Song

**Affiliations:** ^1^ School of Mechanical and Electric Engineering Guangzhou University Guangzhou 510006 China; ^2^ School of Science MOE Key Laboratory for Nonequilibrium Synthesis and Modulation of Condensed Matter Frontier Institute of Science and Technology State Key Laboratory for Mechanical Behaviour of Materials Xi'an Jiaotong University Xi'an 710049 China; ^3^ Shenzhen Institute for Quantum Science and Engineering and Department of Physics Southern University of Science and Technology Shenzhen Key Laboratory of Quantum Science and Engineering Shenzhen Guangdong 518055 China; ^4^ Ferroic Physics Group National Institute for Materials Science Tsukuba Ibaraki 305‐0047 Japan

**Keywords:** classical magnetic system, cluster spin glass, disorder, Griffiths phase, La_2/3_Sr_1/3_(Mn_1‐3x_Al_2x_Ti_x_)O_3_

## Abstract

The presence of Griffiths phase to cluster spin glass transition has theoretically been predicted in both classical and quantum systems. However, its detection in a classical system has been lacking for decades, which hinders a complete understanding of the relationship between the Griffiths phase and cluster spin glass. Here, the experimental discovery of the Griffiths phase to cluster spin glass transition is reported in a classical magnetic system, diluted ferromagnets La_2/3_Sr_1/3_(Mn_1‐3_ *_x_* Al_2_ *_x_* Ti*_x_*)O_3_ (0 ≤ *x* ≤ 0.12). The phase diagram of the system shows a transition from the Griffiths phase into a ferromagnetic state in the low disorder concentration range (0.01 < *x* ≤ 0.09). In the high disorder concentration range (0.09 < *x* ≤ 0.12), a Griffiths phase to cluster spin glass transition is identified, which nicely matches that of disordered quantum systems. Moreover, the Griffiths phase is essentially an unfrozen cluster spin glass with partially broken ergodicity is demonstrated experimentally. These findings serve as crucial experimental references for understanding the glassy phenomena in disordered magnets, facilitating future exploration of their unique properties and functionalities.

## Graphical abstract

Clear experimental evidence of a Griffiths phase to cluster spin glass transition in a classical magnetic system is presented. While this transition is predicted to exist in both classical and quantum systems, but only identified in quantum systems thus far, thereby hindering a complete understanding of the relationship between them. This study provides critical insights for fully understanding of their relationship.

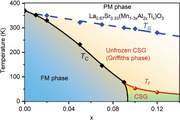

## 1 Introduction

Disorder can induce many exotic phases, such as cluster spin glass (CSG) and the Griffiths phase (GP).^[^ ^1^, ^2^ ^]^ The GP was initially proposed in diluted Ising ferromagnets^[^ ^3^ ^]^ and has its experimental realization been reported in various quantum^[^ ^2^, ^4^, ^5^, ^6^ ^]^ and classical magnetic systems^[^ ^7^, ^8^, ^9^ ^]^ with disorders. The essential characteristic of the GP is the emergence of a local spin ordering, known as the ferromagnetic (FM) cluster, which begins at the onset temperature *T_G_* within the paramagnetic (PM) state and persists down to the Curie temperature *T_C_* of the FM transition. Consequently, the inverse magnetic susceptibility deviates from the Curie–Weiss law within the temperature range *T_C_ < T < T_G_*.^[^ ^3^, ^7^ ^]^ The GP is somewhat similar to the well‐known CSG, showing spin clusters and nonexponential relaxation behaviors^[^ ^10^, ^11^ ^]^; the relationship between the two phases has attracted long‐standing scientific interests.^[^ ^2^ ^]^ Theoretical studies predicted that the GP may transform into CSG in both the classical *d*‐dimensional (*d* > 4 or *d* = 3) lattice^[^ ^10^, ^11^ ^]^ and the quantum magnetic systems.^[^ ^12^, ^13^ ^]^ Early experimental studies have uncovered the quantum GP to CSG transition near absolute zero.^[^ ^14^, ^15^ ^]^ However, no experimental evidence has been reported for the GP to CSG transition for classical magnets, in which the transition may appear at much higher temperatures (several tens K). As a result, a full understanding of the relationship between GP and CSG is still lacking due to the absence of solid experimental evidence of the classical GP to CSG transition.

The classical GP has been frequently reported in the manganites with quenched disorder.^[^ ^7^, ^16^, ^17^, ^18^ ^]^ The coupling among charge, spin, and lattice in these systems leads to the phase complexity and rich phase diagrams,^[^ ^17^ ^]^ which reveal a wide range of ground states such as the FM insulator/metal, orbital‐ and charge‐ordered antiferromagnetic (AFM) phase,^[^ ^19^, ^20^, ^21^ ^]^ glassy‐like phase separation state with coexisting FM and AFM clusters,^[^ ^22^, ^23^ ^]^ and GP.^[^ ^7^, ^17^ ^]^ Moreover, many experimental and theoretical investigations demonstrate that the intrinsic inhomogeneities,^[^ ^17^, ^24^, ^25^ ^]^ GP,^[^ ^18^, ^24^, ^26^ ^]^ dynamical polaron clusters,^[^ ^21^, ^27^, ^28^ ^]^ quantum‐critical behavior^[^ ^18^, ^29^ ^]^ and phase competition^[^ ^21^, ^23^, ^27^, ^29^, ^30^ ^]^ in these systems may give rise to the colossal magnetoresistance (CMR) effect. Notably, the manganites showing GP are primarily the pseudo‐binary solid solution systems with one FM terminal and one AFM terminal on each side. In these systems, the emergence of GP above *T*_C_ in the FM/AFM phase boundary regime is attributed to the competition of FM and AFM phases,^[^ ^17^, ^18^ ^]^ accompanied by orthorhombic to rhombohedral structural transition.^[^ ^7^ ^]^ However, the disorder in these systems is not strong enough to establish the CSG featuring frozen disordered magnetic moments, and the long‐range FM or AFM order is stabilized on further cooling. Given the similarity of ferroic materials, the GP in the ferromagnetic materials has its physical parallel analog — the precursory tweed in ferroelastics and the ergodic relaxor in ferroelectrics. Unlike the case of GP to CSG transition, the classical transitions from precursory tweed to strain glass (ferroelastic glass) or from ergodic relaxor to relaxor (ferroelectric glass) are widely observed^[^ ^31^, ^32^, ^33^, ^34^, ^35^, ^36^, ^37^, ^38^, ^39^, ^40^ ^]^ owing to the strong disorder strength in these material classes. The disorders in these materials are not created by solid soluting two systems with different types of ferroic transitions but by solid soluting one with the ferroic transition and the other without transition, which is equivalent to doping some “non‐transforming” defects into the system with the long‐range ferroic ordering. Such defects lead to very strong disorders, which can destroy the long‐range interactions responsible for magnetic orders and induce a glassy state with short‐range order at low temperatures.

In this work, we adopt a similar material design strategy for ferroelastic and ferroelectric materials in ferromagnetic materials to realize the GP to CSG transition. The La_2/3_Sr_1/3_MnO_3_ was selected as the FM terminal because the GP commonly emerges in these strong‐correlated manganites. The ferromagnetism in La_2/3_Sr_1/3_MnO_3_ stems from the double‐exchange interaction between Mn^3+^ and Mn^4+^ ions.^[^ ^7^ ^]^ We then dilute and disrupt the long‐range magnetic order by substituting nonmagnetic Al^3+^ and Ti^4+^ for Mn^3+^ and Mn^4+^ ions in the same mole ratio. This method is equivalent to the pseudo‐binary solid solution systems with the La_2/3_Sr_1/3_MnO_3_ FM terminal and the La_2/3_Sr_1/3_(Al_2/3_Ti_1/3_)O_3_ nonmagnetic terminal. We systematically studied the behavior of the diluted ferromagnets, La_2/3_Sr_1/3_(Mn_1‐3_ *_x_* Al_2_ *_x_* Ti*_x_*)O_3_ (0 ≤ *x* ≤ 0.12), and constructed the phase diagram. We observed a transition from GP to CSG below a freezing temperature (*T_f_*) in the high disorder regime (*x* > 0.09). Furthermore, we demonstrated that GP is essentially an unfrozen state of CSG with partially broken ergodicity. This study provides experimental evidence for the GP to CSG transition in a classical magnetic system and establishes the unified characteristics of glassy phenomena in ferroic materials.

## 2 Results and Discussion

### 2.1 GP and CSG Transition of La_2/3_Sr_1/3_(Mn_1‐3_ *_x_* Al_2_ *_x_* Ti*_x_*)O_3_

**Figure 1:**
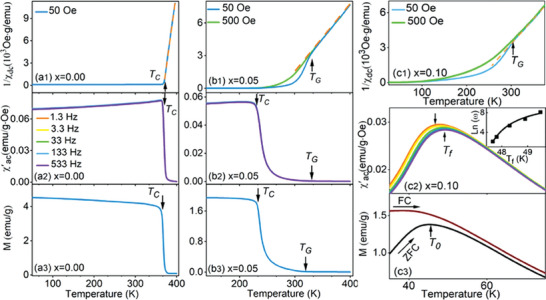
Magnetic properties of three representative La_2/3_Sr_1/3_(Mn_1‐3_ *_x_* Ti*_x_* Al_2_ *_x_*)O_3_ (*x* = 0.00, 0.05, and 0.10) samples. These samples represent the FM terminal (*x* = 0), the low disorder composition showing the GP to FM transition (*x* = 0.05), and the high disorder composition showing the GP to CSG transition (*x* = 0.10). (a1–c1) Inverse DC magnetic susceptibility curves, (b2–c2) AC magnetic susceptibility curve, (b3–c3) and magnetization curves are shown. The 1/*χ*_dc_ of *x* = 0.05, 0.10 in (b1) and (c1) begin to deviate from the Curie–Weiss Law below the GP transition temperature *T_G_*, revealing the PM to GP transition. The *χ*_ac_ peak in (c2) exhibits frequency‐dispersion, while in (c3), the zero‐field‐cooled/field‐cooled (ZFC/FC) magnetization curves for *x* = 0.10 start to deviate at the ZFC peak temperature, *T_0_*, indicating a freezing transition from GP to CSG. The *χ*_ac_ peak temperature (*T_f_*) follows the Vogel–Fulcher relation (inset of (c2)), *ω* = *ω_0_* exp[‐*E_a_*/*k_B_* (*T_f_*‐*T_0_*)], where *ω* is the angular frequency (*ω* = 2π*f*), *ω_0_* is a characteristic attempt frequency, *E_a_* is the activation energy, *k_B_* is the Boltzmann constant, *T_f_* is the spin glass freezing temperature, and *T_0_* is the ideal freezing temperature.

**Figure** **1**a1–a3 shows the evolution of magnetic properties for the FM terminal La_2/3_Sr_1/3_MnO_3_ (*x* = 0). The AC magnetic susceptibility curve (Figure 1a2) displays a sharp step with negligible frequency dependence. Similarly, the magnetization versus temperature (*M‐T*) curve (Figure 1a3) also shows a steep drop around the FM ordering temperature (*T_C_*). The inverse DC susceptibility of the sample is well described by the Curie–Weiss Law (indicated by the orange dashed line) in the entire PM regime above *T_C_*. (Figure 1a1). All these features are typical for a classical FM transition.

After substituting nonmagnetic Ti^4+^ and Al^3+^ ions for Mn^4+^ and Mn^3+^, the AC magnetic susceptibility curve (Figure 1b2) and the *M‐T* curve (Figure 1b3) of La_2/3_Sr_1/3_(Mn_0.85_Al_0.1_Ti_0.05_)O_3_ (*x* = 0.05) retains the typical features of an FM transition around *T_C_* ∼232K. However, the DC inverse susceptibility curve deviates from the Curie–Weiss Law with negative curvature above *T_C_* (Figure 1b1. This deviation signals the development of a GP phase^[^ ^7^, ^8^ ^]^ with an onset temperature of *T_G_* ∼322K representing the GP transition temperature. Moreover, Figure 1b1 shows that the deviation is suppressed with an increased applied magnetic field, consistent with previous observations of GP singularity.^[^ ^7^, ^8^ ^]^ Our magnetic measurements (Figures S5 and S6, Supporting Information) further reveal that the La_2/3_Sr_1/3_(Mn_1‐3_ *_x_* Ti*_x_* Al_2_ *_x_*)O_3_ system undergoes a sequence of transitions PM→GP→FM upon cooling within the low disorder concentration regime (0.01 < *x* ≤ 0.09).

**Figure 2:**
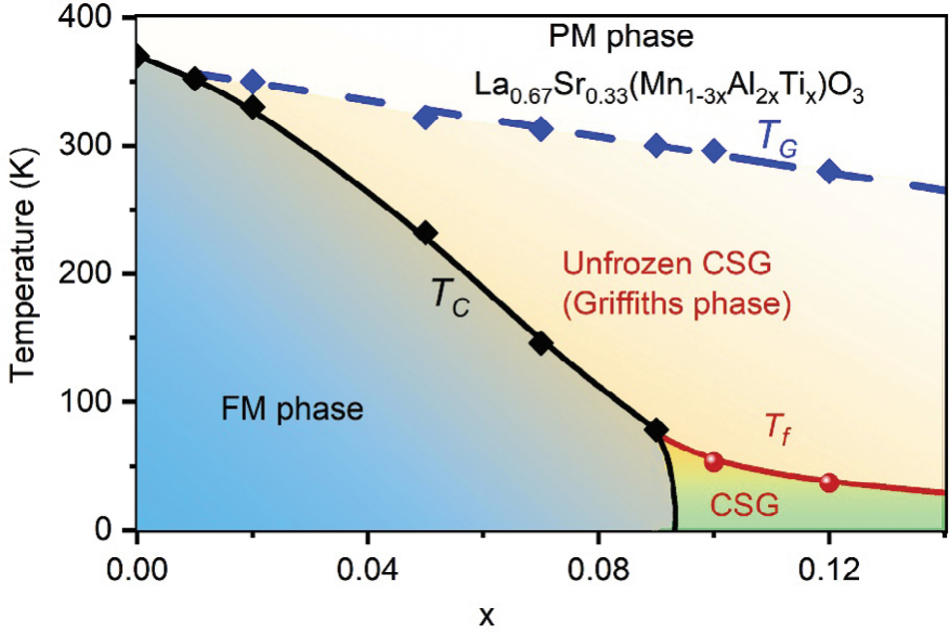
Temperature‐disorder phase diagram of the La_2/3_Sr_1/3_(Mn_1‐3_ *_x_* Ti*_x_* Al_2_ *_x_*)O_3_ (0 ≤ *x* ≤ 0.12) system. The GP transforms into a FM state in the low disorder concentration range (0.01<*x*≤0.09), consistent with the classical GP scenario. However, unlike the classical GP phase diagram,^[^ ^7^ ^]^ the GP changes into a frozen CSG with high disorder concentrations (0.09 < *x* ≤ 0.12). This phase diagram matches well with quantum GP system, disordered ferroelastic and ferroelectric materials.

For a higher disorder concentration, La_2/3_Sr_1/3_(Mn_0.7_Al_0.2_Ti_0.1_)O_3_ (*x* = 0.10), the GP transition is also observed below *T_G_* in the inverse DC susceptibility, as shown in Figure 1c1. Remarkably, a freezing transition indicating the emergence of a CSG occurs below *T_G,_* which has not been reported previously. Two well‐known signatures of a glassy transition are observed for this CSG transition: frequency dispersion in the AC susceptibility and history‐dependence in the static property.^[^ ^32^, ^34^, ^41^, ^42^, ^43^ ^]^ The AC susceptibility curves of La_2/3_Sr_1/3_(Mn_0.7_Al_0.2_Ti_0.1_)O_3_, as shown in Figure 1c2 and Figure S7 (Supporting Information), exhibit frequency‐dependent peaks. The corresponding peak temperature (*T_f_*) follows the Vogel–Fulcher relationship, *ω* = *ω_0_* exp[‐*E_a_*/*k_B_* (*T_f_*‐*T_0_*)]^[^ ^39^, ^40^ ^]^ (inset of Figure 1c2), where *ω* is the angular frequency, *ω_0_* is a characteristic attempt frequency, *E_a_* is the activation energy, and *T_0_* is the “ideal‐glass” temperature. The relaxation time *τ* obtained for *x* = 0.10 and *x* = 0.12 are ≈10^−7^ and 10^−8^ s, respectively, typical for spin glasses; the corresponding *T_0_* values are 46.8 and 36.9 K for *x* = 0.10 and *x* = 0.12, respectively. These findings provide evidence for a dynamic freezing transition, namely, the CSG transition,^[^ ^32^, ^34^, ^43^ ^]^ with *T_f_* being the freezing temperature of the CSG transition.

The history‐dependence of the static magnetization for this sample was detected using zero‐field‐cooling (ZFC)/field‐cooling (FC) measurements, as described in the previous investigation.^[^ ^30^ ^]^ As shown in Figure 1c3, the FC curve increases continuously, while the ZFC curve exhibits a peak around *T_0_* (≈52K) as the temperature decreases. The ZFC/FC curves coincide at high temperatures but deviate upon cooling, eventually showing significant divergences at low temperatures, indicating broken ergodicity and a low‐temperature frozen glass state.^[^ ^36^, ^40^, ^44^ ^]^ As shown in Figure 1, Figures S5–S7 (Supporting Information), the La_2/3_Sr_1/3_(Mn_1‐3_ *_x_* Al_2_ *_x_* Ti*_x_*)O_3_ system undergoes a transition path of PM→GP→CSG upon cooling within the high disorder concentration range (0.09 < *x* ≤ 0.12).

### 2.2 New Phase Diagram of GP and Three Key Features of an Unfrozen “Ferrioc Glass”

#### 2.2.1 New Phase Diagram of GP

The finding of GP to CSG transition in the La_2/3_Sr_1/3_(Mn_1‐3_ *_x_* Ti*_x_* Al_2_ *_x_*)O_3_ system establishes a new phase diagram of GP, as shown in **Figure** **2**. The system undergoes a transition from PM to GP upon cooling below *T_G_* with the disorder concentration of 0.01 ≤ *x* ≤ 0.12. The GP then transforms into a FM state with lower disorder concentrations (0.01 ≤ *x* ≤ 0.09), consistent with the classical GP scenario. With high disorder concentrations (0.09 < *x* ≤ 0.12), the GP undergoes a transition into the frozen CSG, which contrasts sharply with the classical GP phase diagram but matches that of a disordered quantum GP,^[^ ^12^, ^13^ ^]^ as well as disordered ferroelastic and ferroelectric systems.^[^ ^33^, ^39^ ^]^ Such a phase diagram has been extensively studied and well‐established in the other two ferroic material classes, showcasing the novel properties of glass.^[^ ^31^, ^32^, ^33^, ^34^, ^35^, ^36^, ^37^, ^38^, ^39^, ^40^, ^45^ ^]^ However, to our knowledge, a parallel phase diagram in ferromagnetic materials with unfrozen glass to frozen glass transition has not been reported. Based on this new phase diagram shown in Figure 2, we refer to the GP as an “unfrozen CSG.” The percentage of GP of our samples (*x* = 0∼0.0.09) was calculated according to the expression: GP (%) =$\frac{T_{G}-T_{C}}{T_{C}}\;$×100%,^[^ ^46^ ^]^ which increases from 0% to 285% as the disorder concentration (*x*) increases from 0 to 0.09. As shown in Figure S10a (Supporting Information), in comparison with the La_0.7_Sr_0.3_V*_x_* Mn_1‐_ *_x_* O_3_, the GP (%) of La_2/3_Sr_1/3_(Mn_1‐3_ *_x_* Al_2_ *_x_* Ti*_x_*)O_3_ increases much more rapidly with disorder concentration, because the disorder introduced by Al^3+^ and Ti^4+^ leads to much greater suppression of *T_C_* (Figure S10b, Supporting Information) and much more stronger disruption to the long‐range magnetic order. In the following, we experimentally demonstrate that the GP of the La_2/3_Sr_1/3_(Mn_1‐3_ *_x_* Ti*_x_* Al_2_ *_x_*)O_3_ displays three key transforming features of an unfrozen “ferrioc glass” from low (*x* = 0.05) to high (*x* = 0.10) disorder concentrations.

#### 2.2.2 Microstructure Identification of the Short‐Range Orders

**Figure 3:**
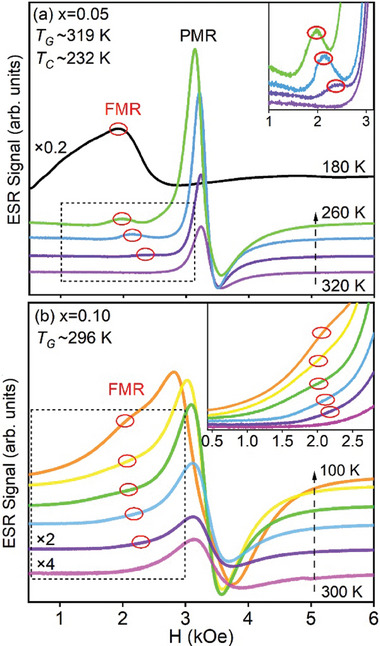
Evolution of ESR spectra with temperatures for La_2/3_Sr_1/3_(Mn_1‐3_ *_x_* Al_2_ *_x_* Ti*_x_*)O_3_ samples with (a) obtained for *x* = 0.05 and (b) for *x* = 0.10. At high temperatures (T≥320K for *x* = 0.05, T≥300K for *x* = 0.10), the ESR spectra exhibit only a paramagnetic resonance (PMR) for both samples. Below the GP transition temperature (*T_G_*), a small ferromagnetic resonance (FMR) signal emerges alongside the PMR, indicating the presence of local FM clusters within the GP of the two samples. The inset of (a) demonstrates that the intensity of the FMR signal of the GP becomes more pronounced as the temperature decreases. In the case of La_2/3_Sr_1/3_(Mn_0.85_Al_0.1_Ti_0.05_)O_3_ (*x* = 0.05) sample (shown in (a)), an FM transition is observed at low temperature, characterized by the disappearance of the PMR signal and the appearance of a large FMR signal below the Curie temperature (*T_C_*).

The presence of short‐range ordering is a crucial characteristic of unfrozen glasses, as observed in ergodic relaxors and precursory tweeds in ferroelectric and ferroelastic systems, respectively.^[^ ^33^, ^34^ ^]^ To microscopically identify the short‐range FM ordering in the GP, we measured ESR spectra for La_2/3_Sr_1/3_(Mn_1‐3_ *_x_* Al_2_ *_x_* Ti*_x_*)O_3_ (*x* = 0.05, 0.07, 0.10), and the results are shown in **Figures** **3** and S11 (Supporting Information). Figure 3a depicts the ESR spectra of La_2/3_Sr_1/3_(Mn_0.85_Al_0.1_Ti_0.05_)O_3_ (*x* = 0.05) obtained at different temperatures, covering both the GP and FM transitions. At high temperatures (T ≥ 320K), the ESR spectrum only displays a PM resonance (PMR) signal at g≈2, which arises from the Mn^3+^/Mn^4+^ spins.^[^ ^6^ ^]^ As the temperature decreases below *T*_G_, another small resonance peak emerges at a lower magnetic field than PMR. This small peak gradually shifts to an even lower field while its intensity increases as temperature decreases (inset of Figure 3a). Eventually, this resonance signal transforms into the FM resonance (FMR) peak below the FM transition temperature (*T_C_*), indicating the formation of local FM clusters within the GP.^[^ ^7^ ^]^ Similarly, Figure 3b and its inset illustrate the evolution of the ESR spectrum for La_2/3_Sr_1/3_(Mn_1‐3_ *_x_* Al_2_ *_x_* Ti*_x_*)O_3_ (*x* = 0.10) across the GP transition from 320K to the lowest measured temperature of 100K. The ESR spectrum of the GP phase also exhibits both a PMR signal and a small FMR signal, indicating the presence of FM clusters. Therefore, the ESR measurements confirm the existence of short‐range FM ordering within the GP of the La_2/3_Sr_1/3_(Mn_1‐3_ *_x_* Al_2_ *_x_* Ti*_x_*)O_3_ system.

#### 2.2.3 The Deviation of Physical Properties for the Short‐Range Order

**Figure 4:**
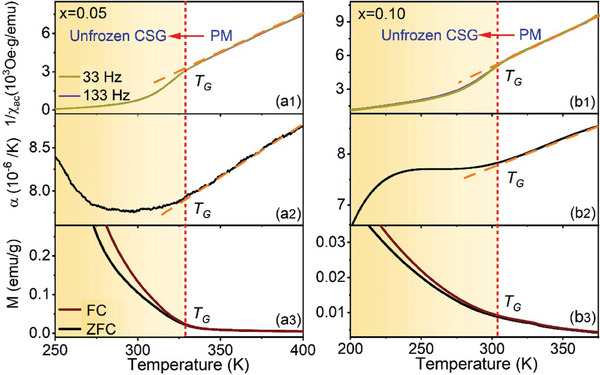
The deviation of physical properties in GP compared to the PM phase is shown in the inverse AC susceptibility, thermal expansion coefficient, and ZFC/FC magnetization curves for La_2/3_Sr_1/3_(Mn_1‐3_ *_x_* Al_2_ *_x_* Ti*_x_*)O_3_ samples with (a1), (a2), and (a3) obtained for *x* = 0.05 and (b1), (b2), and (b3) obtained for *x* = 0.10.

The formation of short‐range order within the unfrozen glass manifests in various physical properties,^[^ ^33^, ^47^ ^]^ such as the ac magnetic susceptibility. The inverse ac susceptibility curves for the La_2/3_Sr_1/3_(Mn_1‐3_ *_x_* Al_2_ *_x_* Ti*_x_*)O_3_ system with *x* = 0.05 and 0.10 are shown in **Figure** **4**a1,b1, respectively. These curves demonstrate that the development of GP leads to deviation from the Curie–Weiss behavior below *T_G_*. Additionally, the normal thermal expansion coefficients for these two samples (Figure 4a2,b2) deviate from that in the PM state below *T_G_*. Such deviations can be attributed to the presence of FM clusters within the PM matrix. The AC susceptibilities and thermal expansion coefficients of the FM clusters are larger than those of the PM matrix, resulting in the observed deviations in the physical properties of the GP.

#### 2.2.4 The Partial Breaking Down of Ergodicity for Unfrozen Glass

Another essential feature of unfrozen glass is the partial breaking down of ergodicity, which can be observed in the ZFC/FC curves. As shown in Figure 4a3,b3, the ZFC/FC curves of the La_2/3_Sr_1/3_(Mn_1‐3_ *_x_* Al_2_ *_x_* Ti*_x_*)O_3_ (*x* = 0.05, 0.10) samples start to bificate at *T_G_*, but they maintain the same tendencies on cooling within the GP regime; this is different from the opposite behavior in the frozen CSG below *T_0_* (Figure 1c3). The characteristic feature of these ZFC/FC curves indicates the partially broken ergodicity in the GP, analogous to the other two unfrozen glasses, such as ergodic relaxors and precursory tweeds.^[^ ^34^, ^36^, ^37^ ^]^

### 2.3 Glassy Dynamics and Possible Formation Mechanism

#### 2.3.1 Glassy Dynamics

**Figure 5:**
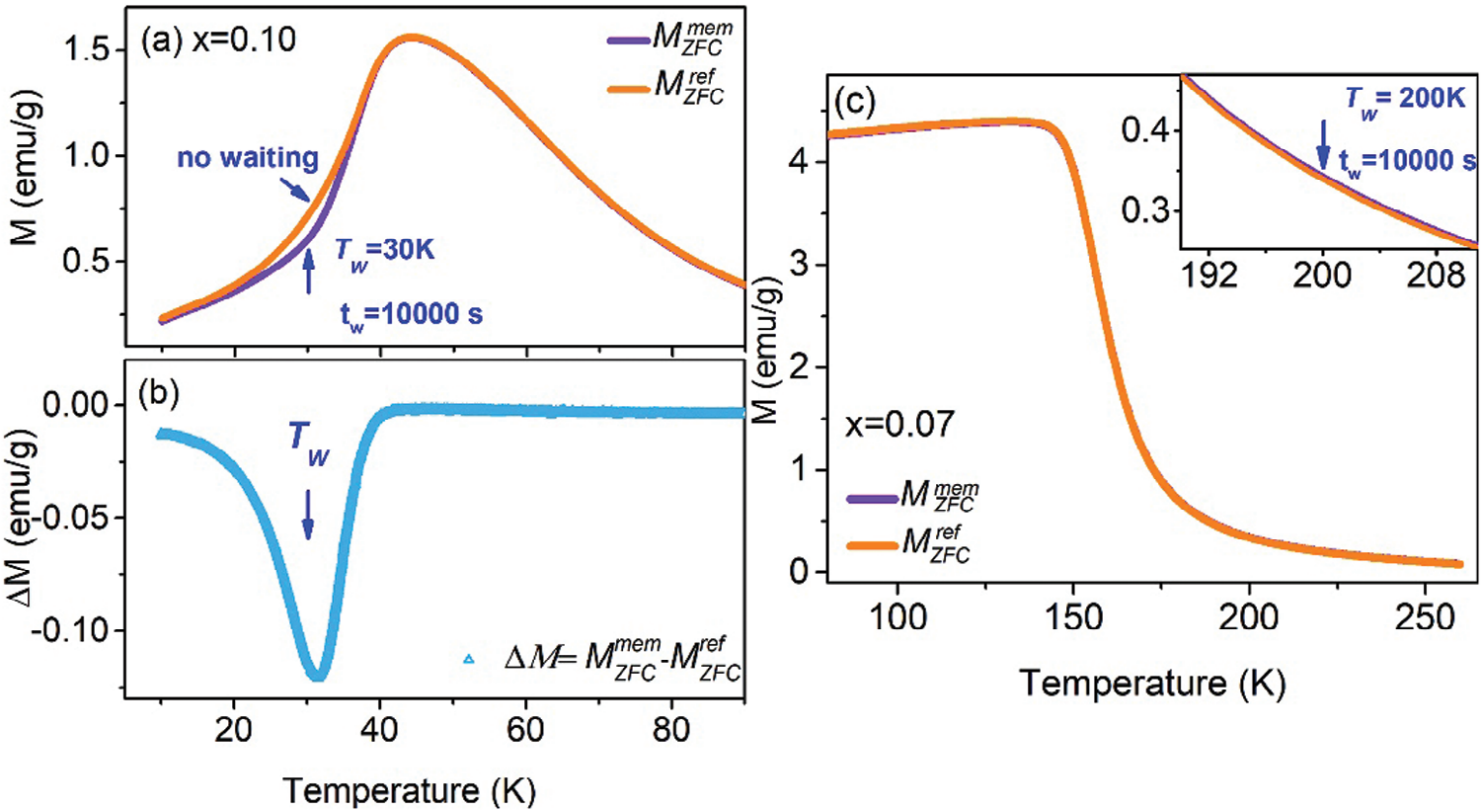
The memory effect of zero‐field‐cooled magnetization (*M_ZFC_*) measured in the CSG phase of La_2/3_Sr_1/3_(Mn_0.7_Al_0.2_Ti_0.1_)O_3_ (*x* = 0.10) and the GP of La_2/3_Sr_1/3_(Mn_0.7_Al_0.2_Ti_0.1_)O_3_ (*x* = 0.07). The magnetization curves 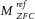 and 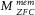 represent the reference and memory curves, respectively. The difference curve △*M* (= 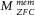 − 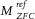) exhibits a memory dip at *T_W_* = 30 K, indicating the presence of a memory effect. However, for *x* = 0.07, there is almost no difference between the 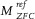 and 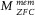 curves. Additionally, there is no decay of magnetization after aging at *T_W_* = 200K in the 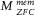 curve (as shown in the inset). These observations suggest the absence of a memory effect in the GP phase.

The nonequilibrium glassy dynamics in the CSG (frozen state) of *x* = 0.10 and the GP (unfrozen state) of *x* = 0.07 were investigated, as depicted in **Figure** **5** (see Supporting Information for a detailed measurement process). We focused on two dynamic effects commonly observed in glassy systems: the memory effect and aging.^[^ ^33^, ^48^ ^]^ The waiting temperatures *T_W_* of aging were selected as 30 K for *x* = 0.10 and 200 K for *x* = 0.07, because *T_W_* = 30 K and *T_W_* = 200 K are located in the CSG temperature region (*T_W_* <*T_g_*) of *x* = 0.10 and the GP temperature region (*T_C_* < *T_W_* <*T_G_*) of *x* = 0.07, respectively. We measured the ZFC magnetization (*M_ZFC_*) to examine the memory effect. The memory ZFC magnetization curve (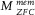), displays a distinct dip around *T_W_* (the waiting temperature), contrasting the reference ZFC magnetization curve (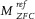). The difference curve △*M* (= 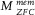 − 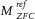) exhibit a memory dip at *T_W_* = 30 K, indicating a strong “memory” of the cooling aging history and highlighting the metastable nature of the spin glass. In contrast, for *x* = 0.07, there was almost no difference between 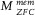 and 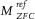 at *T_w_* = 200 K. This behavior suggests the absence of a memory effect in the GP due to its unfrozen nature.

#### 2.3.2 Possible Formation Mechanism

For magnetic materials with certain forms of disorder, the competition of two factors governs their transforming behaviors.^[^ ^49^ ^]^ One is the global effect, namely, the thermodynamic driving force of FM transition that stabilizes the long‐range FM order. The other is the local effect caused by the disorder, which generates frustrations and simultaneously suppresses the long‐range FM order but promotes the formation of short‐range FM order. The appearance of high‐temperature GP results from the local effect of the disorder. The local effect dictates local FM clusters in the dynamically disordered PM matrix, leading to the partial breaking down of ergodicity. The global effect becomes stronger with a decrease in temperature because the system tends to form long‐range FM order to reduce entropy. For the low disorder concentration range, the local effect is weaker than the global effect at low temperatures; the FM thermodynamic driving force can drive the GP to change to FM. However, for the high disorder concentration range, the local effect dominates the global effect at low temperatures. Thus, the FM state is not able to form. Instead, frustration makes the randomly distributed local FM clusters gradually freeze into CSG on cooling, accompanying the global breaking down of ergodicity.

## 3 Conclusion

In summary, we observed the GP by constructing diluted ferromagnets, La_2/3_Sr_1/3_(Mn_1‐3x_Al_2x_Ti_x_)O_3_. Notably, a freezing transition from GP to CSG was observed in the highly disordered regime, providing the first experimental evidence for the glassy transition of GP in the classical system. The newly established phase diagram is consistent well with quantum GP system, disordered ferroelastic and ferroelectric materials. With further measurement of the corresponding physical properties analogous to ferroelastics and ferroelectrics, we demonstrated that GP is essentially an unfrozen state of CSG with partially broken ergodicity. This discovery opens up new avenues of research in conventional spin glass. It potentially leads to the exploration of intriguing properties, just as strain glass or relaxor have done in the past.

## 4 Experimental Section

### Material Preparations

La_2/3_Sr_1/3_(Mn_1‐3_ *_x_* Ti*_x_* Al_2_ *_x_*)O_3_ (0 ≤ *x* ≤ 0.12) samples were fabricated using a solid‐state reaction method with high purity (99.9%) La_2_O_3_, Mn_2_O_3_, SrCO_3_, TiO_2_, and Al_2_O_3_. The samples were calcined at 1073—1373 K for 3 h and then sintered at 1573 K for 3 h. To prevent Mn volatilization, the samples were sintered in closed crucibles and covered with excess powder with the same composition.

### Measurement of Physical Properties

Magnetic measurements were conducted in a superconducting quantum interference device (SQUID) from Quantum Design. The temperature evolution of the local FM clusters was monitored using an X‐band (≈9.1 GHz) electron spin resonance (ESR) system (JOEL, JES‐FA200). Moreover, the thermal expansion behavior was measured using a thermomechanical analyzer (Netzsch, TMA 402 F2) at a 2 K min^−1^ cooling rate. The structure and valence states of La_2/3_Sr_1/3_(Mn_1‐3_ *_x_* Al_2_ *_x_* Ti*_x_*)O_3_ are determined by X‐ray powder diffraction (XRD) and X‐ray photoelectron spectroscopy (XPS) measurements (Figure S1 and Figures S2–S4, Supporting Information).

### Memory Effect Analysis

The memory effect of GP and CSG was analyzed by measuring the zero‐field‐cooled magnetization (*M_ZFC_*) after typical stop‐and‐wait protocols.^[^ ^50^ ^]^ The waiting temperatures *T_W_* were selected as 30 K for *x* = 0.10 and 200 K for *x* = 0.07, because they are located in the CSG temperature region (*T_W_* <*T_g_*) of *x* = 0.10 and the GP temperature region (*T_C_* < *T_W_* <*T_G_*) of *x* = 0.07, respectively. The 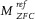 was recorded during heating at the 3 K min^−1^ in H = 50 Oe after continuous ZFC process, i.e., the standard *M_ZFC_* procedure. While the 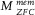 was measured in same warming process, but with intermediate stop‐and‐wait cooling at *T_W_* = 30 K for *x* = 0.10 and *T_W_* = 200 K for *x* = 0.07 with aging time *t_w_* = 10 000 s.

## Conflict of Interest

The authors declare no conflict of interest.

## Supporting information



Supporting Information

## Data Availability

The data that support the findings of this study are available from the corresponding author upon reasonable request.
