# Supplementary material for: Evidence for a Griffiths Phase to Cluster Spin Glass Transition in the La2/3Sr1/3(Mn1‐3 x Al2 x Ti x )O3 System
Source: Adv Sci (Weinh). 2024 Oct 14;11(45):2408517. doi: 10.1002/advs.202408517 (PMC11615757; doi:10.1002/advs.202408517)
Supplement: Supplementary file 1 — Supporting Information [file ADVS-11-2408517-s001.docx]

Supporting Information

**Evidence for a Griffiths Phase to Cluster Spin Glass Transition in the La_2/3_Sr_1/3_(Mn_1-3_*_x_*Al_2_*_x_*Ti*_x_*)O_3_ System**

*Ruie Lu, Yuanchao Ji, Yu Wang,** *Xiaoqin Ke, Fanghua Tian, Chao Zhou, Yin Zhang, Chang Liu, Sen Yang,** *Xiaobing Ren, and Xiaoping Song*

Figure S1 presents the XRD patterns of La_2/3_Sr_1/3_(Mn_1-3_*_x_*Al_2_*_x_*Ti*_x_*)O_3_ (*x* = 0.00, 0.05, 0.10). XRD analysis shows that all three samples exhibit the rhombohedral structure^[1]^, indicating that the structure of La_2/3_Sr_1/3_(Mn_1-3_*_x_*Al_2_*_x_*Ti*_x_*)O_3_ unchanged after Al^3+^ and Ti^4+^ doping, with no impurities present in the La_2/3_Sr_1/3_(Mn_1-3_*_x_*Al_2_*_x_*Ti*_x_*)O_3_ system.

**
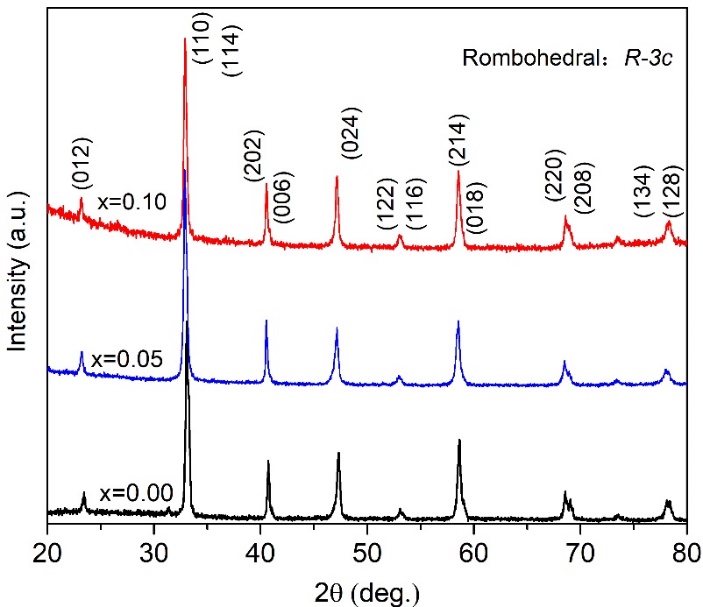
**

**Figure S1.** XRD spectrum of La_2/3_Sr_1/3_(Mn_1-3_*_x_*Al_2_*_x_*Ti*_x_*)O_3_ with *x* = 0.00, 0.05, 0.10.

To investigate the O-stoichiometry and valence states of elements in the La_2/3_Sr_1/3_(Mn_1-3_*_x_*Al_2_*_x_*Ti*_x_*)O_3_ system, we conducted X-ray photoelectron spectroscopy (XPS) measurement. The wide-range-surface scan XPS spectra of La_2/3_Sr_1/3_MnO_3_ and core-level XPS spectra of La_2/3_Sr_1/3_(Mn_1-3_*_x_*Al_2_*_x_*Ti*_x_*)O_3_ (*x* = 0.0, 0.05, 0.10) are shown in figures. S2-S4. The surface of the material contains elements such as La, Sr, Mn, and O ((Figure S2(a)), and the C impurity at 284.8 eV is due to surface absorption during air exposure. To calibrate, carbon correction was applied using the C1s line positioned at 284.8 eV to mitigate any charging effect errors.

Figures. S2-S4 show the core-level XPS spectra of La3d, Sr2p, Mn2p, Al2p, Ti2p, and O1s, with all peaks matched to the National Institute of Standards and Technology database. The Mn 2p spectra (figure S2(d), S3(c), and S4(c)) display two main peaks of 2p_3/2_ and 2p_1/2_, resulting from spin-orbit coupling ^[2, 3]^. The XPS peak fitting analysis of Mn 2p_3/2_ and Mn 2p_1/2_ confirms the presence of two mixed valence states^[4]^. The peaks observed at approximately 641.3 and 652.8 eV correspond to the Mn 2p_3/2_ and Mn 2p_1/2_ of Mn^3+^ ions, respectively, and the peaks at approximately 642.4 and 654.7 eV correspond to the Mn 2p_3/2_ and Mn 2p_1/2_ of Mn^4+^ ions, respectively. Notably, the intensity ratio of the Mn^3+^ and Mn^4+^ peaks is close to 2, confirming that the O-stoichiometry in La_2/3_Sr_1/3_(Mn_1-3_*_x_*Al_2_*_x_*Ti*_x_*)O_3_ is equivalent to +3.


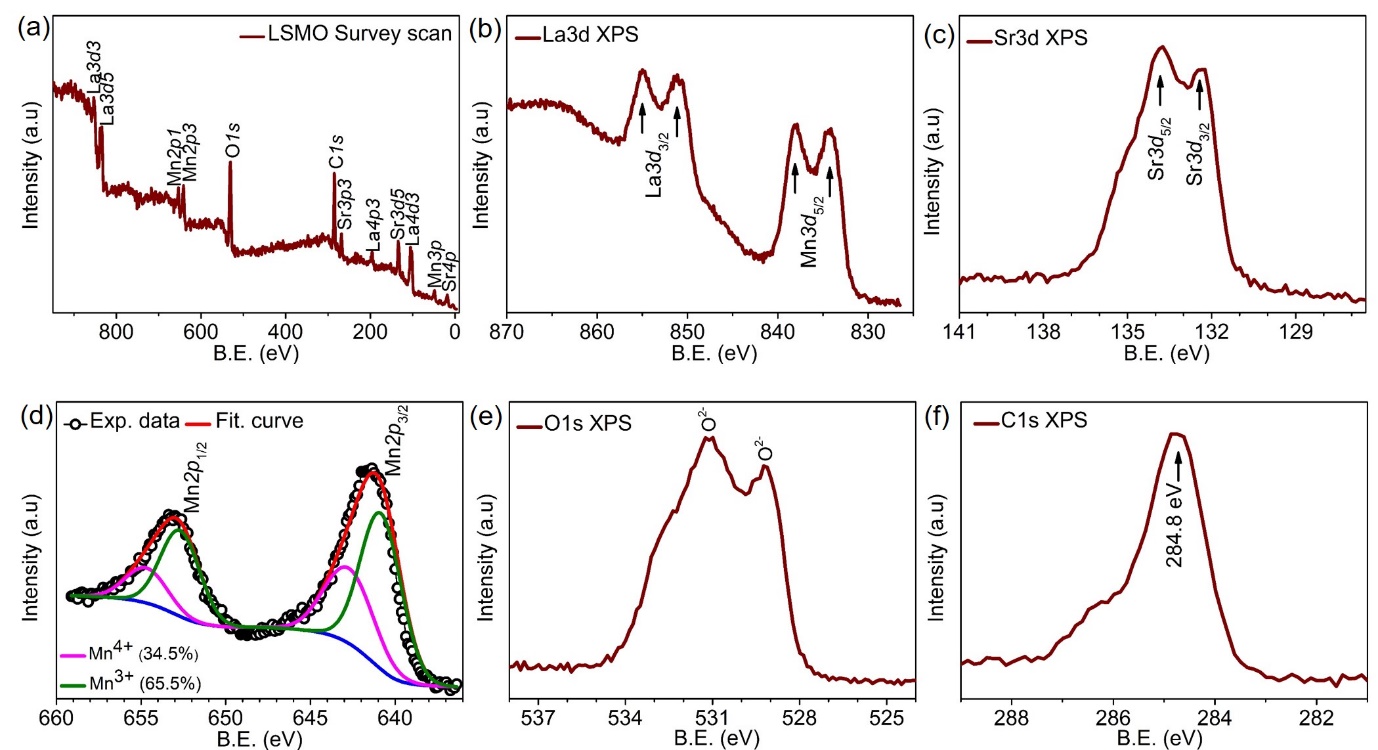


**Figure S2.** (a-f) Wide-range-surface scan and core-level X-ray photoemission spectroscopy (XPS) of La, Sr, Mn, O, and C elements present on the surface of La_2/3_Sr_1/3_MnO_3_ (*x* = 0).


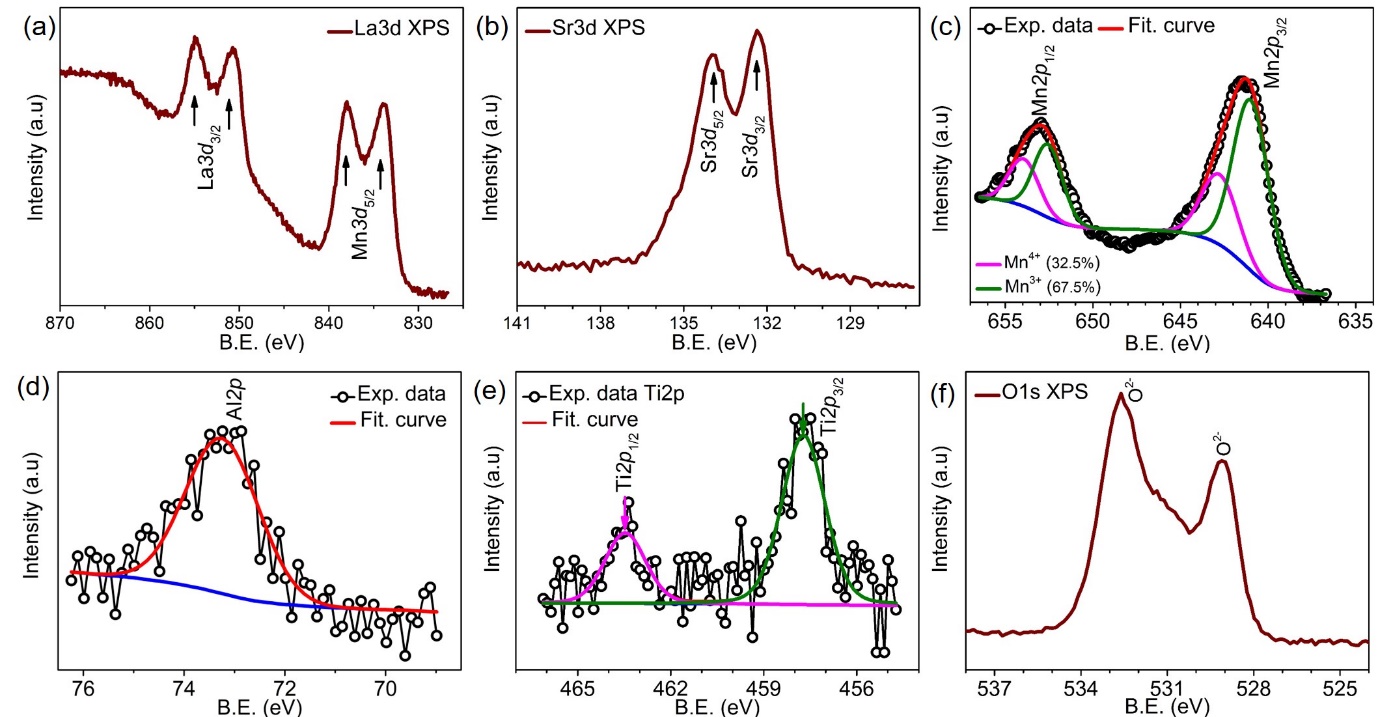


**Figure S3.** (a-f) Core-level XPS of La, Sr, Mn, Al, Ti, and O elements on the surface of La_2/3_Sr_1/3_(Mn_0.85_Al_0.1_Ti_0.05_)O_3_ (*x* = 0.05).


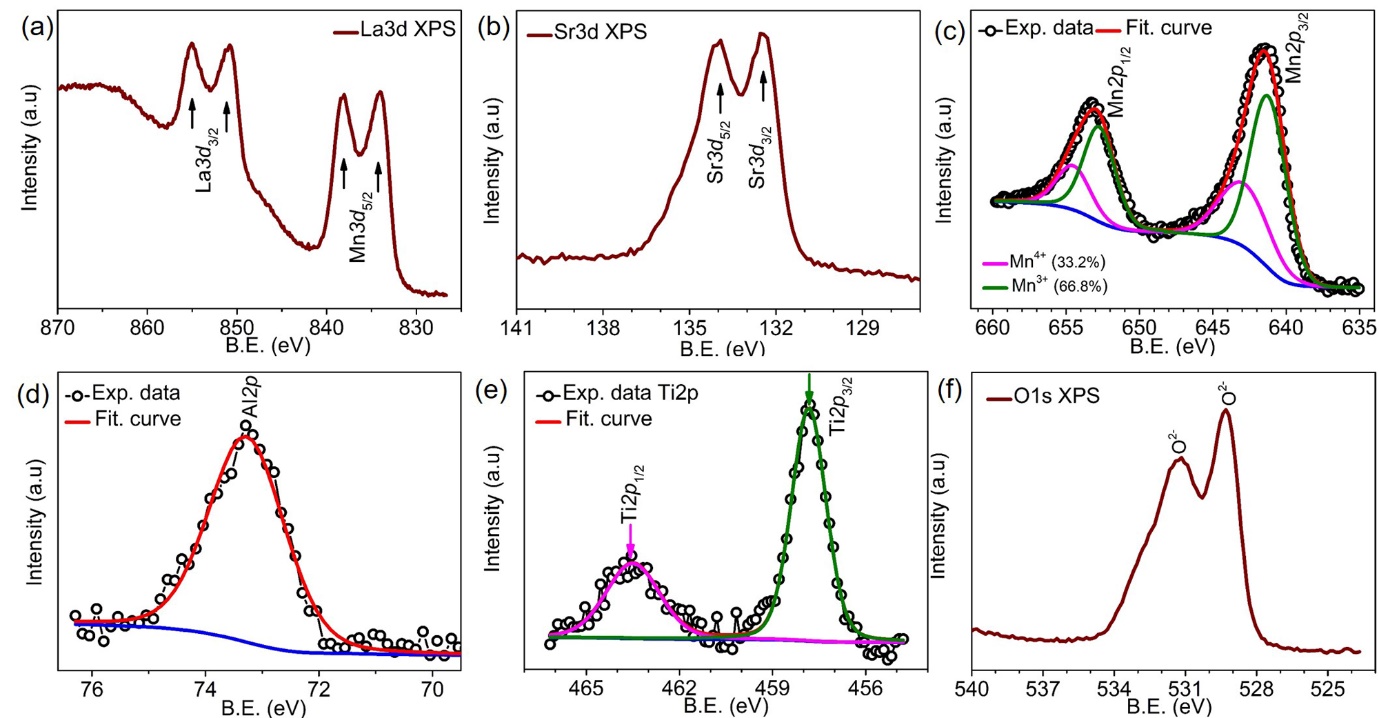


**Figure S4.** (a-f) Core-level XPS of La, Sr, Mn, Al, Ti, and O elements on the surface of La_2/3_Sr_1/3_(Mn_0.85_Al_0.2_Ti_0.10_)O_3_ (*x* = 0.10).

The XPS spectrum of La3d (figures S2(b), S3(a), and S4(a)) exhibits two main peaks of 3d_5/2_ and 3d_3/2_, each splitting into two partially resolved peaks. This observation is consistent with the findings in Ref. [5]. The Sr3d spectrum (figures S2(c), S3(b), and S4(b)) also shows two peaks of 3d_5/2_ and 3d_3/2_ with a splitting of 1.6 eV ^[6]^. The Al2p spectrum (figures S3(d) and S4(d)) at 73.3 eV corresponds to the Al^3+^ state in La_2/3_Sr_1/3_(Mn_1-3_*_x_*Al_2_*_x_*Ti*_x_*)O_3_ systems and is consistent with the Al2p spectrum observed in LaAlO_3_ ^[7]^. The Ti2p_3/2_ and Ti2p_1/2_ peaks at 457.8 and 463.6 eV (figures S3(e) and S4(e)), respectively, indicate the presence of Ti^4+^ ^[8]^. The O1s XPS spectra (figures S2(e), S3(f), and S4(f)) display a doublet due to the presence of two types of oxides in La_2/3_Sr_1/3_(Mn_1-3_*_x_*Al_2_*_x_*Ti*_x_*)O_3_, namely LaMnO_3_ and LaAlO_3_ ^[9]^. Combining XPS analyses with the XRD results leads to the confirmation that the La_2/3_Sr_1/3_(Mn_1-3_*_x_*Al_2_*_x_*Ti*_x_*)O_3_ system is considered “clean” and follows the expected stoichiometry.


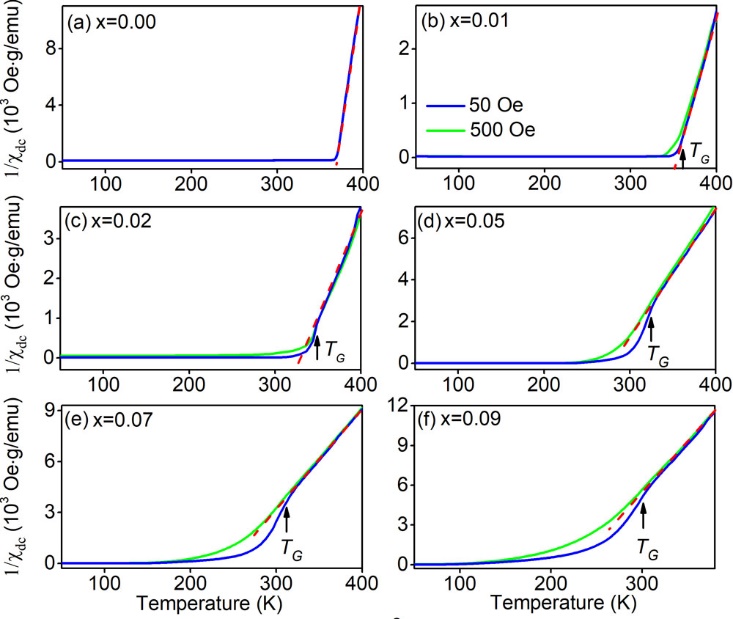


**Figure S5.** Temperature dependence of inverse DC magnetic susceptibility for La_2/3_Sr_1/3_(Mn_1-3_*_x_*Al_2_*_x_*Ti*_x_*)O_3_ (*x* = 0.00, 0.02, 0.05, 0.07, 0.09, 0.10) samples. *T_G_* is the GP transition temperature, where *1*/*χ_dc_* begins to deviate from the Curie–Weiss law (indicated by the red dashed line). Below *T_G_*, *1*/*χ_dc_* shows a strong field dependence, consistent with previous observations of GP. It reveals that the La_2/3_Sr_1/3_(Mn_1-3_*_x_*Ti*_x_*Al_2_*_x_*)O_3_ system undergoes a sequence of transitions PM🡪GP🡪FM upon cooling within the low disorder concentration regime (0.01 < *x* ≤ 0.09).


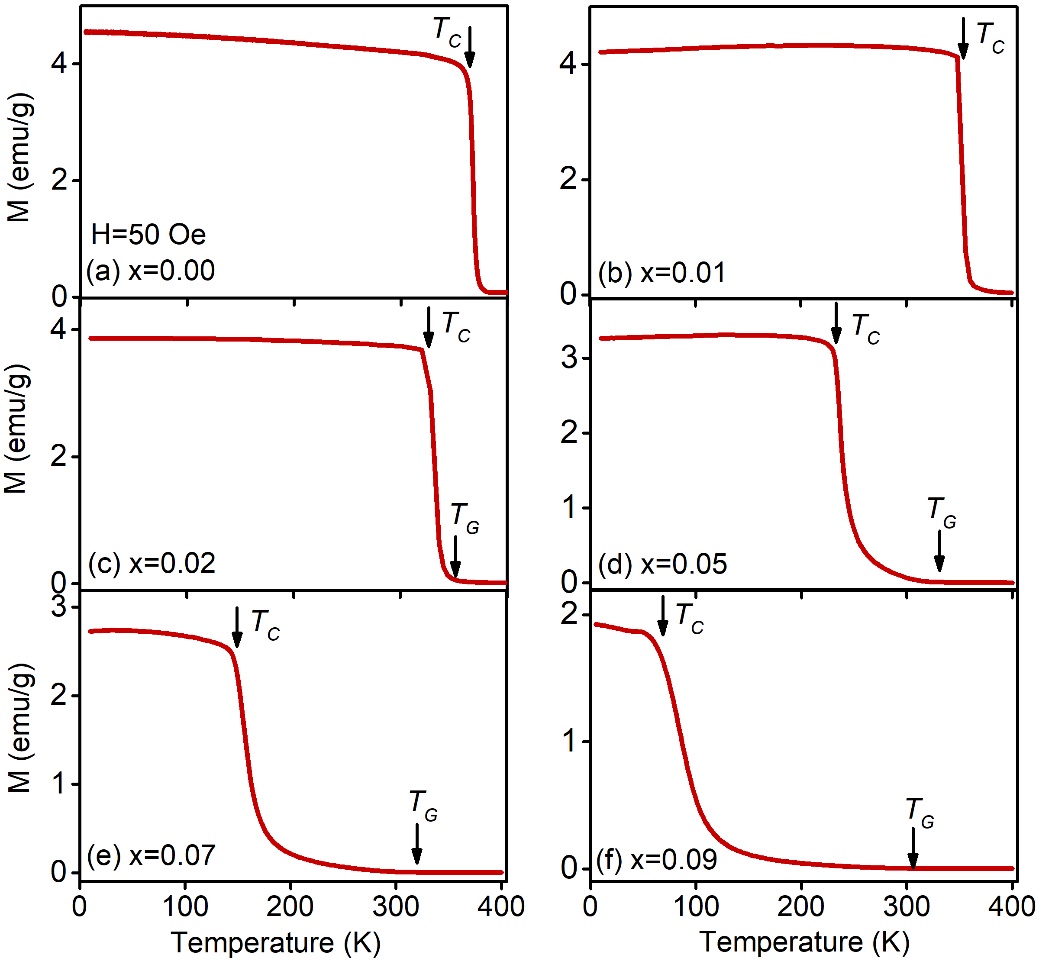


**Figure S6.** Magnetization vs. temperature curves under *H*=50 Oe after the same field cooling (FC) process for La_2/3_Sr_1/3_(Mn_1-3_*_x_*Al_2_*_x_*Ti*_x_*)O_3_ (*x* = 0.00, 0.02, 0.05, 0.07, 0.09, 0.10) samples. The (*M*–*T*) curves show a steep drop around the FM ordering temperature (*T_C_*). The *T_C_* values for each composition are as follows: *T_C_* = 370, 352, 330, 232, 146, and 78 K.


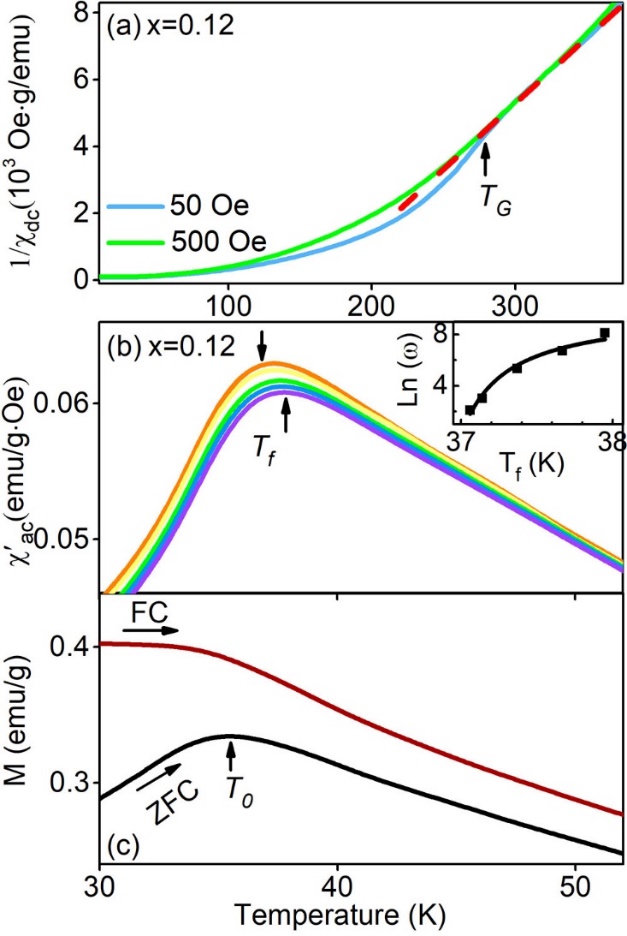


**Figure S7.** GP and CSG transitions were observed for a high disorder concentration La_2/3_Sr_1/3_(Mn_0.64_Al_0.24_Ti_0.12_)O_3_ (*x* = 0.12). Upon cooling, the system initially transforms from a PM state to the GP, where *1*/*χ_dc_* begins to deviate from the Curie–Weiss law, and then to a CSG transition at a freezing temperature *T_f_*, at which the AC susceptibility shows frequency-dependent peaks with the peak temperature following the Vogel–Fulcher relation (inset), *ω* = *ω_0_*exp[-*E_a_*/*k_B_*(*T_f_*-*T_0_*)], where *ω* is the angular frequency, *ω_0_* is a characteristic attempt frequency, *E_a_* is the activation energy, *k_B_* is the Boltzmann constant, *T_f_* is the spin glass freezing temperature, and *T_0_* is the “ideal-glass” temperature. Additionally, the CSG transition is reflected by a large deviation between the ZFC and FC magnetization curves below *T_0_* (the peak temperature in the ZFC curve).


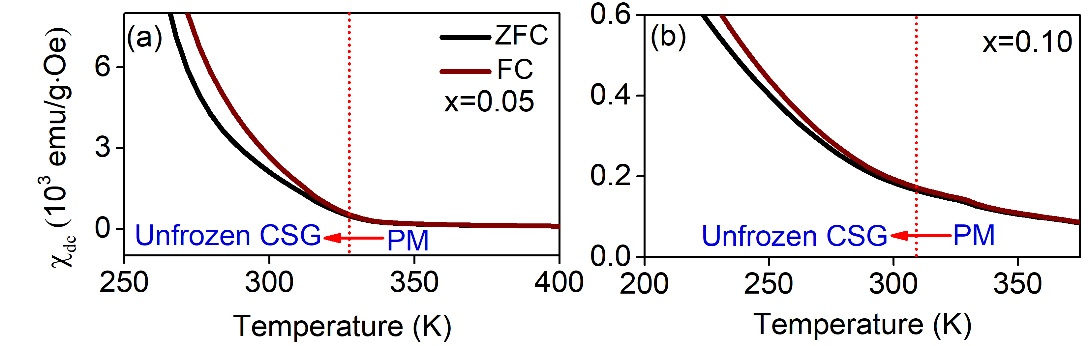


**Figure S8.** DC susceptibility ZFC/FC curves around *T_G_* for La_2/3_Sr_1/3_(Mn_1-3_*_x_*Al_2_*_x_*Ti*_x_*)O_3_ (*x* = 0.05, 0.10) samples.

Figure S9 shows the in-plane (*χ*´) and out-plane (*χ*ʺ) ac susceptibility for the FM, GP, and CSG transitions. The *χ*ʺ curve exhibits a sharp peak with frequency independence, and the *χ*´ curve shows a steep jump at *T_C_* (figures S9(a1) and (a2)) indicating the onset of FM ordering. For the GP transition, the *χ*ʺ curve displays a frequency-independent step (figures S9(b2) and (c3)), and the *χ*´ curve starts to increase (inset of figures S9(b1) and (c3)). This suggests the formation of magnetic clusters in the GP. During the CSG transition at *T_f_*, both the *χ*´ and *χ*ʺ curves exhibit frequency dispersion (figure S9(c1) and (c2). This suggests that the dynamic nature of the CSG that the relaxation processes within the CSG are influenced by the measurement frequency.


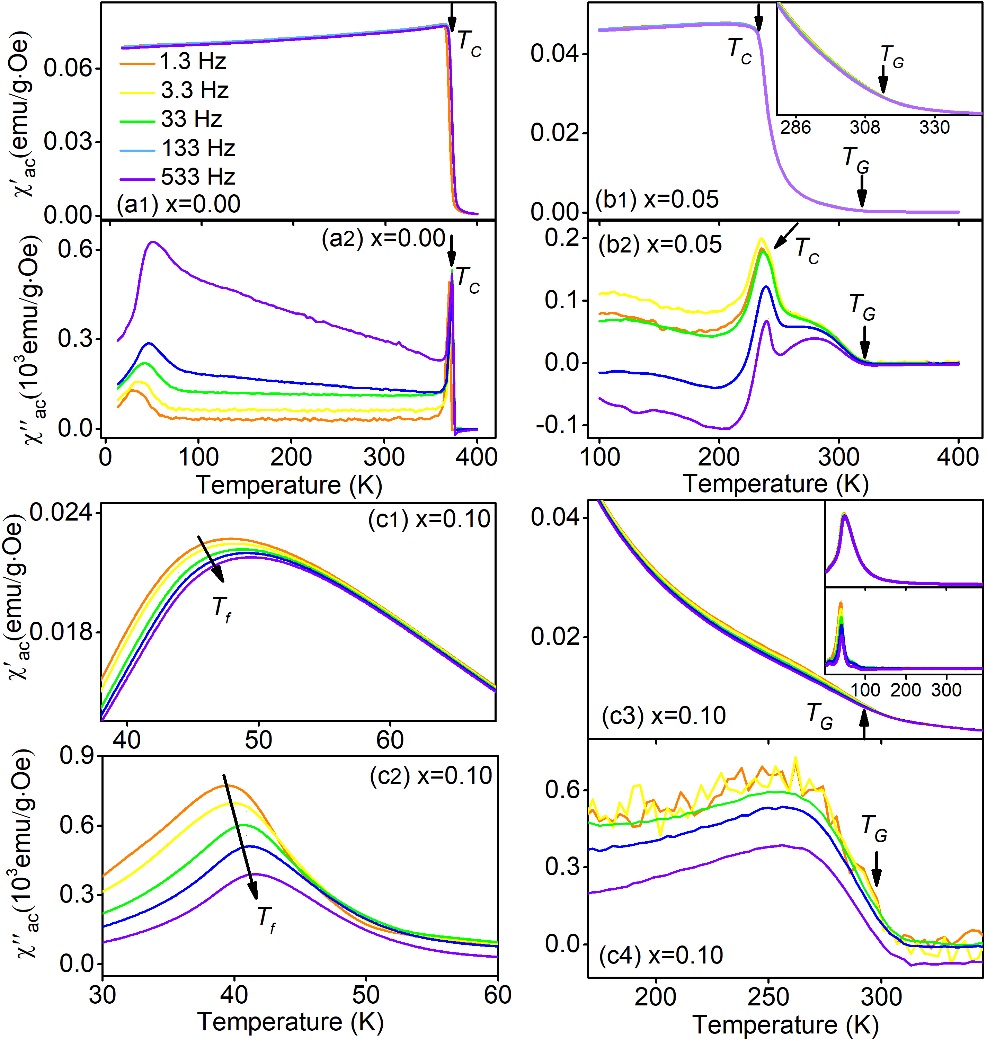


**Figure S9.** In-plane (*χ*´) and out-of-plane (*χ*ʺ) AC susceptibility of three representative La_2/3_Sr_1/3_(Mn_1-3_*_x_*Ti*_x_*Al_2_*_x_*)O_3_ (*x* = 0.05, 0.10) samples at the specific critical temperatures *T_G_*, *T_C_*, and *T_f_*. Inset (b1) shows the *χ*´ of *x* = 0.05 around *T_G_*. Insets (c3) show the *χ*´ and *χ*ʺ curves over the temperature range of 5-400 K.


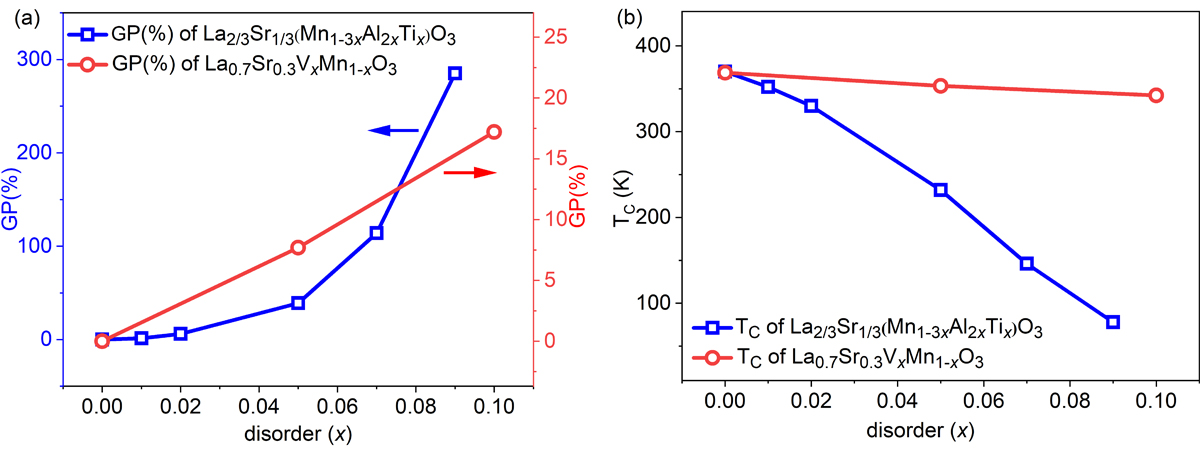


**Figure S10.** (a) Proportion of Griffiths phase GP(%) and (b) Curie temperature *T_C_* as the function of disorder concentration *x* for our sample La_2/3_Sr_1/3_(Mn_1-3_*_x_*Al_2_*_x_*Ti*_x_*)O_3_ and the similar compound La_0.7_Sr_0.3_V*_x_*Mn_1-_*_x_*O_3_ reported previously ^[10]^.


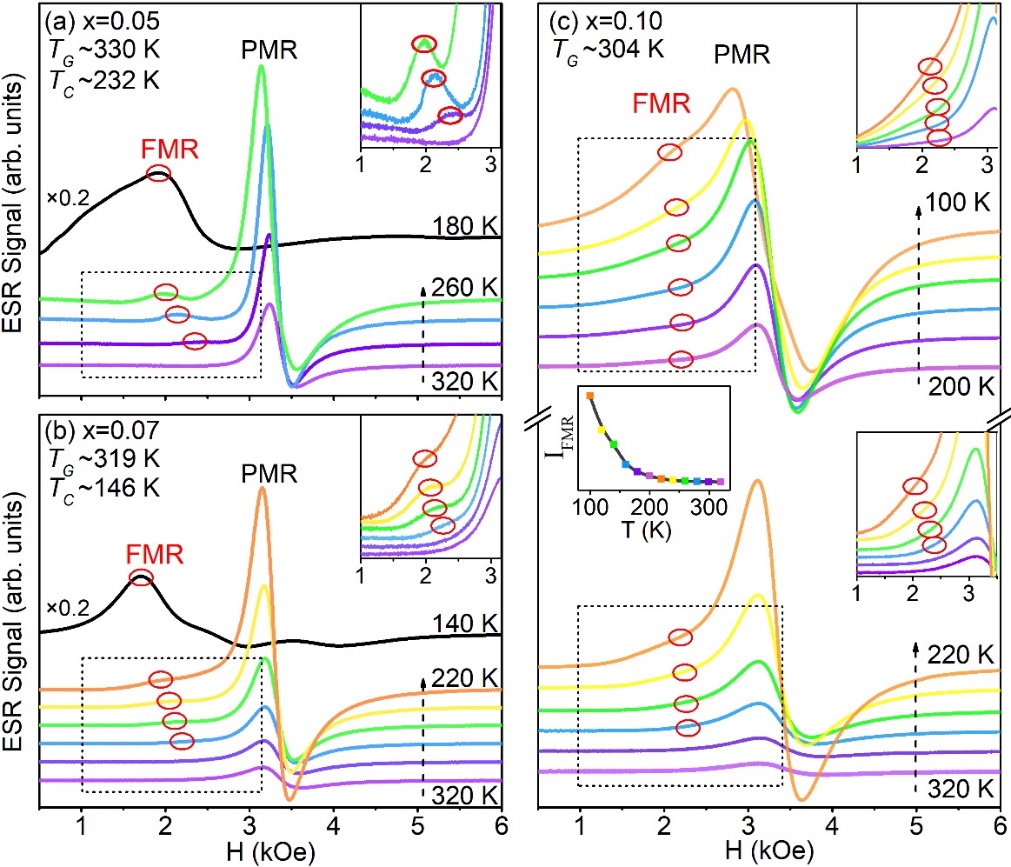


**Figure S11.** Evolution of ESR spectra with temperatures for La_2/3_Sr_1/3_(Mn_1-3_*_x_*Al_2_*_x_*Ti*_x_*)O_3_ (*x* = 0.05, 0.07, 0.10) samples. Below *T_G_*, another smaller FMR peak emerges at a lower magnetic field than PMR. This suggests the formation of local FM clusters within the GP. The FMR peak gradually shifts to a lower field while its intensity increases as temperature decreases (see insets), suggesting a gradual growth of these FM clusters. The ESR results confirm the existence of short-range FM ordering within the GP of La_2/3_Sr_1/3_(Mn_1-3_*_x_*Al_2_*_x_*Ti*_x_*)O_3_ systems.

References

[1] S. M. Zhou, Y. Li, Y. Q. Guo, J. Y. Zhao, X. Cai, L. Shi, *J. Appl. Phys.* **2013**, *114*, 163903.

[2] K. Anand, A. Pal, P. Singh, M. Alam, A. G. Joshi, A. Mohan, S. Chatterjee, *arXiv:1910.13734* **2019**.

[3] M. Alam, A. Pal, K. Anand, P. Singh, S. Chatterjee, *arXiv:1909.12599* **2019**.

[4] G. D. Dwivedi, S. Kumar, A. G. Joshi, S. Kumar, A. K. Ghosh, H. Chou, H. D. Yang, S. Chatterjee, *J. Alloys Compd.* **2017**, *699*, 31.

[5] L. Schlapbach, H. R. Scherrer, *Solid State Commun* **1982**, *41*, 893.

[6] R. P. Vasquez, *Surface Science Spectra* **1992**, *1*, 112.

[7] R. P. Vasquez, *Surface Science Spectra* **1992**, *1*, 361.

[8] F. B. Li, X. Z. Li, M. F. Hou, *Applied Catalysis B: Environmental* **2004**, *48*, 185.

[9] N. Gunasekaran, S. Rajadurai, J. J. Carberry, N. Bakshi, C. B. Alcock, *Solid State Ionics* **1994**, *73*, 289.

[10] J. K. G, A. Jose, E. P. Jinu, T. T. Saravanan, E. Senthil Kumar, M. Navaneethan, H. Sreemoolanadhan, K. Kamala Bharathi, *J Phys D Appl Phys* **2022**, *55*, 215001.
